# Obsessive-compulsive disorder comorbid with rheumatoid arthritis: case report and review of literature

**DOI:** 10.1192/j.eurpsy.2023.1959

**Published:** 2023-07-19

**Authors:** H. S. Amani, M. Rim

**Affiliations:** Psychiatry A departement, Hedi Chaker university hospital Sfax Tunisia, sfax, Tunisia

## Abstract

**Introduction:**

Obsessive-compulsive disorders are complex pathologies causing a major psychosocial handicap. However, their association with a disabling somatic pathology such as rheumatoid arthritis makes management more difficult.

**Objectives:**

To investigate through a case analysis and a review of literature the association between obsessive-compulsive disorder (OCD) and rheumatological disorders.

**Methods:**

We reported a case of a woman with a long history of obsessive compulsive disorder who presented rheumatoid arthritis and we conducted a review of literature through search on Pub-Med/MEDLINE following the terms “obsessive-compulsive disorder”, “rheumatoid arthritis”, “association”, “inflammation”.

**Results:**

Case presentation: A 62-years old woman who had been followed at the psychiatric consultation for 20 years for OCD. She had been stabilised on clomipramin at a dose of 100 mg per day until 2012 and since then she had been lost to follow-up with the notion of poor compliance with the treatment. She re-consulted in August 2022 for worsening psychiatric symptoms such as phobic obsessions with delusional beliefs, verification compulsions, sleep disorders and multiple somatic complaints including diffuse arthralgia and chronic arthritis evolving for 6 months.

The patient was put on risperidone 2mg with anxiolytic without improvement. She was referred to the rheumatology consultation where the diagnosis of very active rheumatoid arthritis was retained, hence she was put on 20mg of prednisolone per day with methotrexate.

The evolution was marked by the accentuation of obsessions and compulsions with the appearance of depressive elements, hence the introduction of fluoxetine 40 mg per day, the increase in the dose of risperidone to 4 mg with the anxiolytic with an improvement on the somatic and psychiatric symptoms.

**Conclusions:**

Literature had shown that patients with this OCD usually suffer from inflammatory or rheumatological comorbidities. This association could complicate the management of these patients.

**Disclosure of Interest:**

None Declared

